# Multifocality and multicentricity are not contraindications for sentinel lymph node biopsy in breast cancer surgery

**DOI:** 10.1186/1477-7819-4-79

**Published:** 2006-11-20

**Authors:** Alberta Ferrari, Paolo Dionigi, Francesca Rovera, Luigi Boni, Giorgio Limonta, Silvana Garancini, Diego De Palma, Gianlorenzo Dionigi, Cristiana Vanoli, Mario Diurni, Giulio Carcano, Renzo Dionigi

**Affiliations:** 1 Department of Surgical Sciences, University of Insubria, Varese, Italy; 2 Department of Nuclear Medicine, University of Insubria, Varese, Italy; 3 Department of Radiology, University of Insubria, Varese, Italy; 4 Dipartimento di Scienze Chirurgiche, Rianimatorie-riabilitative e dei Trapianti d'Organo, University of Pavia, Fondazione IRCCS Policlinico San Matteo, Pavia, Italy

## Abstract

**Background:**

After the availability of the results of validation studies, the sentinel lymph node biopsy (SLNB) has replaced routine axillary dissection (AD) as the new standard of care in early unifocal breast cancers. Multifocal (MF) and multicentric (MC) tumors have been considered a contraindication for this technique due to the possible incidence of a higher false-negative rate. This prospective study evaluates the lymphatic drainage from different tumoral foci of the breast and assesses the accuracy of SLNB in MF-MC breast cancer.

**Patients and methods:**

Patients with preoperative diagnosis of MF or MC infiltrating and clinically node-negative (cN0) breast carcinoma were enrolled in this study. Two consecutive groups of patients underwent SLN mapping using a different site of injection of the radioisotope tracer: a) "2ID" Group received two intradermal (ID) injections over the site of the two dominant neoplastic nodules. A lymphoscintigraphic study was performed after each injection to evaluate the route of lymphatic spreading from different sites of the breast. b) "A" Group had periareolar (A) injection followed by a conventional lymphoscintigraphy. At surgery, both radioguided SLNB (with frozen section exam) and subsequent AD were planned, regardless the SLN status.

**Results:**

A total 31 patients with MF (n = 12) or MC (n = 19) invasive, cN0 cancer of the breast fulfil the selection criteria. In 2 ID Group (n = 15) the lymphoscintigraphic study showed the lymphatic pathways from two different sites of the breast which converged into one major lymphatic trunk affering to the same SLN(s) in 14 (93.3%) cases. In one (6.7%) MC cancer two different pathways were found, each of them affering to a different SLN. In A Group (n = 16) lymphoscintigraphy showed one (93.7%) or two (6.3%) lymphatic channels, each connecting areola with one or more SLN(s). Identification rate of SLN was 100% in both Groups. Accuracy of frozen section exam on SLN was 96.8% (1 case of micrometastasis was missed). SLN was positive in 13 (41.9%) of 31 patients, including 4 cases (30.7%) of micrometastasis. In 7 of 13 (53.8%) patients the SLN was the only site of axillary metastasis. SLNB accuracy was 96.8% (30 of 31), sensitivity 92.8 (13 of 14), and false-negative rate 7.1% (1 of 14). Since the case of skip metastasis was identified by the surgeon intraoperatively, it would have been no impact in the clinical practice.

**Conclusion:**

Our lymphoscintigraphic study shows that axillary SLN represents the whole breast regardless of tumor location within the parenchyma. The high accuracy of SLNB in MF and MC breast cancer demonstrates, according with the results of other series published in the literature, that both MF and MC tumors do not represent a contraindication for SLNB anymore.

## Background

During the last few years world-wide consensus has been obtained for SLNB as new standard of axillary staging in early breast cancer. According to the International Consensus Conference on SLNB (Philadelphia, 2001) [1] the technique is indicated for unifocal, infiltrating and clinically node-negative breast carcinoma up to 3 cm in diameter. Absolute or relative contraindications include pregnancy, tumors downstaged by neoadjuvant chemotherapy, previous breast or axillary surgery, some cases of multifocality (once total diameter of the breast quadrant involved by cancer is greater than 3 or 5 cm) and multicentricity [1].

Recently, both European and United States guidelines on breast cancer treatment have included SLNB as first choice option for early infiltrating, unifocal and clinically node-negative lesions, providing that such procedure is performed by experienced surgeons [2-4]. However, several limits for application of this technique have been recently revised [5] in order to extend the potential benefit of avoiding unnecessary axillary dissection. The role of SLNB in multifocal (MF) and multicentric (MC) breast cancer is one of the most common topic open to debate.

Although the terms MF and MC have often been used to identify the same condition, MC cancer should be defined as multiple synchronous tumors originating in different sites of the breast, and MF cancer as multiple foci of the same tumor.

Over the past few years, several clinical and anatomical criteria have been proposed to identify MF and MC cancers, including the distance between cancer, foci (greater than 2, 3 or 5 cm in MC cancer), the presence of histologically normal tissue among nodules (MC), the location in the same (MF) or different (MC) quadrants of the breast [6,7].

During early diffusion of the SLNB technique the main theory of lymphatic drainage of the breast postulated the presence of multiple pathways connecting different sites of the breast to different SLNs. As consequence, both MF and MC breast cancers were considered relative (MF) or absolute (MC) contraindications to SLNB [1] due to concerns about the possibility to identify the "true" SLN. Moreover, earlier results of validation studies on SLNB technique suggested the possible incidence of a higher false-negative rate in MF and MC cancers [8]. Nevertheless, recent studies support now a different theory about breast lymphatic drainage: the SLN (one or more SLNs) would be representative of the whole breast, considered as a single limphatic unit [9,10]. In this case, SLNB technique in MF and MC cancers should be as accurate as in unifocal breast tumors [11].

This prospective study was designed to evaluate the lymphatic drainage from different tumoral foci of the breast and to assess the accuracy of the SLNB in MF and MC invasive breast cancers.

## Patients and methods

Patients with preoperative diagnosis of MF or MC breast carcinoma were prospectively enrolled in this study. Cases of suspicious MF and MC tumors identified after clinical, mammographic and echographic assessment had to be confirmed by positive fine needle aspiration cytology (FNAC) or core biopsy histology in at least two of the nodules; cases with one positive lesion associated with other suspicious nodules with athypical cells on FNAC were also included in the study.

Breast cancer was defined as MF if two or more lesions were located in the same quadrant and distance from each other less than 5 cm. If nodules arises in different quadrants of the breast and/or were distant each other more than 5 cm the cancer was defined as MC. Exclusion criteria were: ductal or lobular *in situ *carcinomas, clinical and/or echographic evidence of positive axilla, neoadjuvant chemotherapy, previous breast or axillary surgery or radiotherapy. Although tumor size greater than 3 or 5 cm is considered a contraindication for SLNB in most guidelines, T2–T3 cancers were included in this study due to recent reports of high accuracy of SLNB even in these cases [12].

Written informed consent was required to include patients in this study and approval from the Ethical Committee of the Hospital was obtained.

The day before surgery SLN mapping was performed using the radioisotope technique [13]. All patients were injected with 20–40 mBq of ^99m^Tc-nanocolloid (< 100 nm in diameter), but in two consecutive groups of patients a different site of injection was used. **1) *2ID Group***. A former series of patients was designed to receive two consecutive intradermal (2ID) injections of the tracer [14] in the cutaneous projections corresponding to the deep site of each nodule (in patients with more than two nodules, injections were performed over the two dominant lesions most distant to each other). In case of non-palpable tumor the technique of radio-guided localization (ROLL) under ultrasonography (US) or stereotaxis was used. During the procedure, the cutaneous projection of the non-palpable lesions was also marked on the skin in order to guide the intradermal injection of the tracer during SLN mapping.

After each tracer injection a lymphoscintigraphic study was planned to evaluate the route of lymphatic spreading from different sites of the breast. The imaging protocol included: 1) early imaging (after 5 minutes) showing the lymphatic route from the site of injection to the SLN; 2) late imaging (after 30 minutes) demonstrating the presence and the number of the SLN(s) identified. Both images were performed with shelded injection site and after positioning of a low activity ^57^Co-flood source in order to obtain the body outline. The lymphoscintigraphic images performed after both the first and the second intradermal injections were then compared in order to recognized if the identified lymphatic route and SLN(s) were coincident or not. **2) *A Group***. In the subsequent group of women included in this study a periareolar (A) injection technique as described by other authors [15] was used, followed by a preoperative lymphoscintigraphy with early (5 minutes) and late (30 minutes) imaging.

Whenever possible, breast surgery was conservative in patients with MF cancer, while, in the remaining cases of MF and in all MC tumors, formal mastectomy with immediate reconstruction was performed. Axillary surgery consisted in the SLNB performed by radioguided technique, followed by AD. Each hot SLN (lymph nodes with counts of > 10 times that of the background counts) was excised and immediately examined with consecutive 200 μm frozen sections and, regardless from the result of SLN status, routine axillary dissection was performed in all patients.

Definitive histopathological examination was performed on SLNs by routine hematoxylin-eosin staining completed by immunohistochemistry for cytokeratin just in doubtful cases. According to the revised TNM staging system of the American Joint Cancer Commission, the SLN was considered positive when a metastatic focus > 0.2 mm in diameter was found on frozen sections and/or on definitive histopathology examination.

If preoperative MF or MC invasive breast cancer diagnosis was not confirmed on the surgical specimen examination, patients were excluded from the study.

## Results

Between January 2004 and December 2005, 292 patients underwent breast surgery for cancer at the Department of Surgery of the University of Insubria. Among these women, 35 patients had preoperative diagnosis of infiltrating MF or MC breast carcinoma; 2 cases were excluded from the study due to clinically positive axilla, confirmed by ultrasound.

The remaining 33 patients were enrolled in the study, and other 2 patients were excluded at the definitive pathologic examination, due to a suspected second focus of ductal carcinoma *in situ *(DCIS) in one patient and atypical ductal hyperplasia (ADH) in the other one.

The remaining 31 patients (all female, mean age 64.1 ± 11.6) had confirmed MF (n = 12) or MC (n = 19) infiltrating cN0 breast carcinoma. The first 15 patients of this series received a double intradermal radioisotope injection over the two most distant neoplastic nodules (2ID Group), while the remaining 16 patients underwent periareolar injection (A Group).

### Lymphoscintigraphic anatomy

In the 2ID Group the lymphoscintigraphic study after each injection showed the pattern of lymphatic spreading from two different sites of the breast. In 14 (93.3%) cases (4 MF, 10 MC) the (*tratto più distale*) pathway of diffusion and the affering SLN(s) were common.

**Figure 1:**
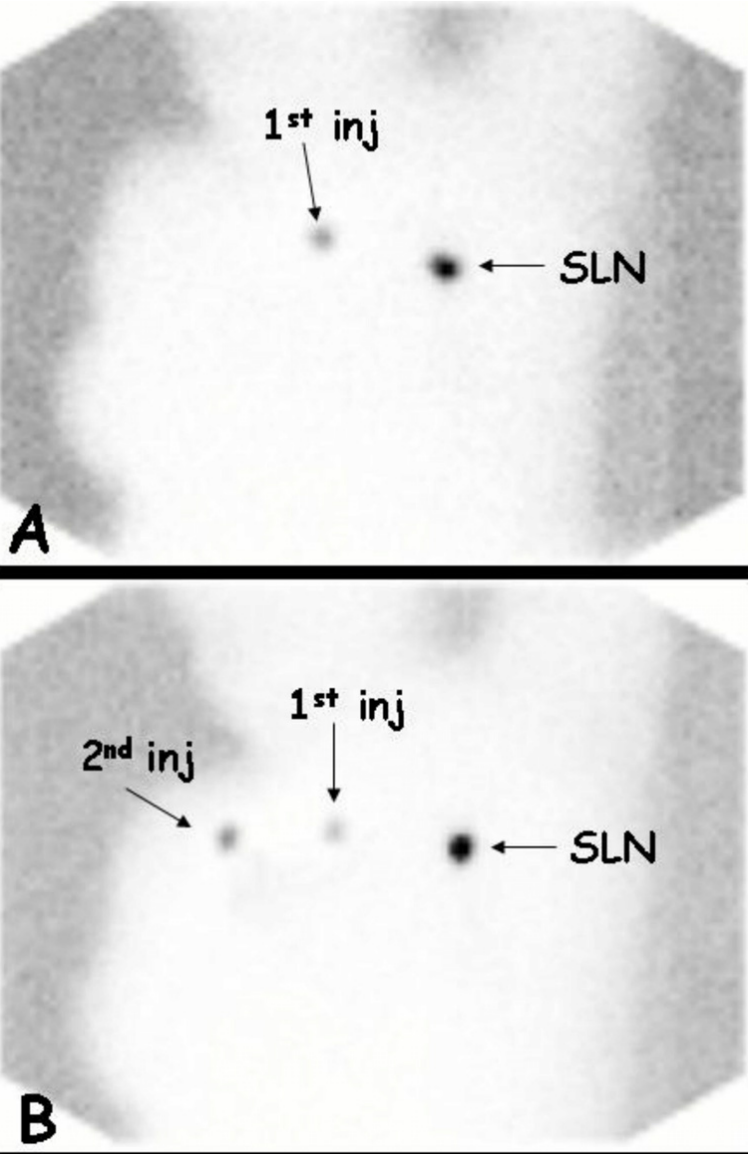
Lymphoscintigraphic study performed in a patient affected by invasive MC breast cancer (two nodules located in the upper outer and inner quadrants of the left breast). The patient underwent two subsequent lymphoscintigraphies after each intradermal injection of the tracer over the two neoplastic foci (2ID Group), showing one sentinel lymphatic channel affering to the same SLN. **A) **First lymphoscintigraphy performed after the first radioisotope injection over the tumoral focus located in the upper outer quadrant of the breast. One SLN is visualized in the axilla. **B) **Second lymphoscintigraphy performed in the same patient after the second radioisotope injection over the other tumoral focus located in the upper inner quadrant of the breast. The same but hotter SLN is visualized in the axilla.

**Figure 2:**
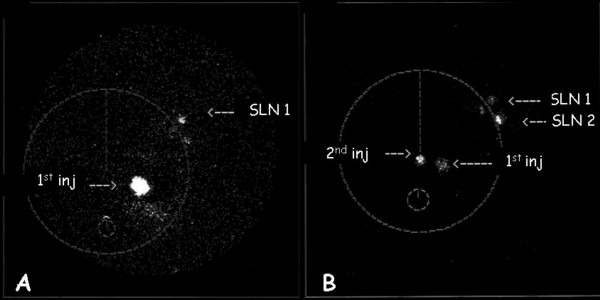
Lymphoscintigraphic study performed in a patient affected by invasive MC breast cancer (two nodules located in the upper outer and inner quadrants of the left breast). The patient underwent two subsequent lymphoscintigraphies after each intradermal injection of the tracer over the two neoplastic foci (2ID Group), showing two sentinel lymphatic channels mainly affering to different SLNs. **A) **First lymphoscintigraphy performed after the first radioisotope injection over the tumoral focus located in the upper outer quadrant of the breast. One SLN is visualized in the axilla. An other LN shows low radioactivity. **B) **Second lymphoscintigraphy performed in the same patient after the second radioisotope injection over the other tumoral focus located in the upper inner quadrant of the breast. One different pathway is visualized traversing the outer upper quadrant of the breast, which is mainly connected to the second SLN visualized after the first injection.

As shown in figure 1, the tracer, even when injected in different quadrants of the breast, migrated in the upper outer quadrant were converged into a unique lymphatic channel which terminated in one or more SLN(s). In only one (6.7%) case of MC cancer two different dominant pathways were present along the upper outer quadrant of the breast (figure 2), each of them affering to a different SLN.

In the A Group lymphoscintigraphy showed one main lymphatic trunk connecting subareolar plexus to one or more SLN(s) in 15 out of 16 cases (93.7%), while in one patient two different lymphatic channels started from areola and each one ended separately at a different SLN.

### Breast tumors features

In 31 patients affected by MF (n = 12) or MC (n = 19) infiltrating breast cancer the number of lesions at preoperative assessment were 2 in 28 cases, 3 in 2 patients and 4 in the remaining one, while histology on surgical specimens revealed 2 lesions in 23 cases, 3 in 6 cases and 4 in 2 cases. Mean diameter of the largest nodule was 23.3 ± 10.1 (10 ÷ 60) mm. Patients were classified as: T1, T2 and T3 in 15 (48.4%), 15 (48.4%) and 1 (3.2%) cases, respectively.

Breast conserving surgery was performed with radical margins in 8 patients with MF cancer, while the remaining 74.2% of cases (4 MF and all 19 MC tumors) underwent mastectomy with immediate reconstruction (except for 3 patients who refused plastic surgery). After SLNB, three levels AD was performed in all patients (mean number of lymph nodes excised per patient: 17.9 ± 6.2).

### Accuracy of SLNB in MF-MC tumors

Using radioisotope technique SLN was identified in all 31 cases (100% identification rate) in both groups. The mean number of SLNs detected was 2.1 ± 0.9 (1–4), without any difference between the 2ID (2.1 ± 0.9) and A Group (2.06 ± 0.9). Histology of SLN from frozen sections was confirmed at definitive pathologic exam in all but one patient (96.8% accuracy rate), in which micrometastasis in the SLN was revealed at definitive histopathological exam alone.

At least one positive SLN was found in 13 (41.9%) out of 31 patients with MF-MC invasive breast cancer: 9 macrometastases (> 2 mm in size) and 4 (30.7%) micrometastases (unique neoplastic focus from 0.2 to 2 mm in size). No case of isolated tumoral cells (ITC) was detected.

In 7 out of 13 (53.8%) patients with positive SLN (including all 4 cases of micrometastatic SLN), such a node was the only site of metastases. SLN status correctly predicted complete axillary status in 30 out of 31 patients; indeed in one patient with 2 negative SLNs, one positive first level lymph node (metastatic focus measuring 11 mm in diameter) was demonstrated after complete AD. However, this positive non radioactive lymph node had been already identified by the surgeon as strongly suspicious for skip metastasis [16,17].

Including this case as a false negative (FN) result, the FN rate was 7.1% (1 out of 14), and sensitivity, negative predictive value and accuracy of SLNB in MF-MC invasive breast cancer were 92.8%, 94.4% and 96.8%, respectively.

## Discussion

During the last years the SLNB technique has replaced routine AD as standard of care for lymph node staging of breast cancer [18]. Excellent accuracy of the technique with low false negative rate is well established in small, unifocal, clinically node-negative tumors, in absence of contraindications such as previous breast or axillary surgery, neoadjuvant chemotherapy, chest-wall irradiation.

To date, SLNB represents a minimally invasive, highly accurate method of axillary staging, which allows almost 65–70% patients to be spared from AD and its related morbidity [19]. Patients affected by MF and MC breast carcinoma are excluded from SLNB in most international guidelines, due to concerns about a possible multiple pattern of lymphatic spreading from different neoplastic nodules of the breast. However, searching for the best technique of SLN mapping stimulated further developments in the field of functional lymphatic anatomy [20].

Historically, under the nipple-areola complex a rich lymphatic network (Sappey plexus) seems to receive the lymph from the whole breast and then drains to the axilla [21], while according to studies from others the lymphatics in the gland are directly connected to the axilla [22]. Although controversies remain about the course of lymph flow between breast parenchyma and axilla, the theory of the breast considered as a single lymphatic unit, at least for what concerns axillary SLN, it is now widely accepted.

The correspondence of lymphatic drainage between the deep glandular and the overlying skin in the same quadrant of the breast has been already demonstrated [23]. Such a "lobary" theory of lymphatic anatomy allowed the diffusion of the intradermal injection of the marker for SLN identification as the best option technique because of the optimal identification rate of SLN [14,16].

More recently, a correspondence between peritumoral and areolar injection has also been demonstrated. Regardless from tumor location within the breast, in over 90% of cases the periareolar injection technique detects the same SLN(s) which is identified by the peritumoral injection using two different markers (radioisotope and blue dye) [9,15,24,25]. Furthermore, also the use of areolar injection techniques as compared to the classical peritumoral provides better results in terms of identification rates of SLN and equal or even better accuracy [26-28].

Lymphoscintigraphic study of Kern *et al*., [29] gives the anatomic support to the "whole breast" theory of axillary lymphatic drainage, demonstrating a single (91%) main lymphatic route (the so-called "sentinel lymphatic channel") draining from the rich areolar plexus to the SLN.

If the deep lymphatic drainage leads to the same SLN(s) of the overlying skin, and the common pathway of this superficial plexus is represented by a sentinel lymphatic channel connecting the areola to the axilla, the SLN should be considered representative of the whole breast, thus overcoming concerns about the accuracy of the technique in case of MF and MC breast cancers.

Although the existence of a sentinel lymphatic channel has been demonstrated by Kern *et al*., by using the areolar injection technique [29], the present study gives the first evidence that intradermal radioisotope injections in two different quadrants of the breast give the same SLN visualization in most of the cases. Furthermore, the lymphoscintigrapic study performed in the present series demonstrates in both 2ID and A groups a common final pathway of the superficial lymphatic plexus of the breast toward the axilla in over 90% of cases, regardless the site of injection.

The common lymphatic pathway of drainage theory for the whole skin envelope of the breast can explain the similar results in term of high accuracy of SLNB in MF and MC cancer reported in this and others series [30-38].

As shown in Table 1, despite of high variability among different studies, all retrospective series [34-36] and multicentric trials [32,37,38] report high accuracy and low false negative rates (< 10%) of SLNB in MF and MC breast cancer. Tousimis *et al*., reported a false negative rate of 8%, falling to 0% once T3 tumors and intraoperative palpable axillary disease (according to our experience) are excluded [17].

**Table 1:** Summary of validation studies of SLNB in MF-MC breast tumors published in the Literature, 1999–2006

**Author**	**year**	**Study**	**n. pts**	**Mapping technique**	**ID %**	**FN %**	**ACC %**
Mertz^30^	1999	Prospective	16	A*	98	0	100
Schrenk^31^	2001	Prospective	19	A^blue ^+/- A*	100	0	100
Kim^11^	2002	Case reports	5	1ID* + T^blue^	100	nv	nv
Fernandez^32^	2002	Multicentric trial	53	T*^+blue ^or ID*^+blue ^or A*^+blue^	98	0	100
Ozmen^33^	2002	Prospective	21 MF	T^blue^	85,7	33,3	77,8
Kumar^34^	2003	Retrospective	59 (48 AD)	T^blue ^+ 1–2ID*	93,5	0	100
Tousimis^35^	2003	Retrospective	70	T*^+blue^	95,9	8	96
Kumar^36^	2004	Retrospective	10 (8 AD)	T* or A*^+blue^	100	0	100
Goyal^37^	2004	Multicentric trial	75 (AD or S)	T*^+blue^	94,6	8,8	95,8
Knauer^38^	2006	Multicentric trial	150 (125 AD)	ns (* or/+^blue^)	ns	4,1	97,4
Current study	2006	Prospective	31	2ID* or A*	100	7,1	96,8

A = areolar injection of radioisotope (A*) or blue dye (A^blue^)

ID = intradermal injection of radioisotope (ID*) or blue dye (ID^blue^) over one (1ID) or two (2ID) neoplastic foci

T = peritumoral injection of radioisotope (T*) or blue dye (T^blue^)

ns = non specified

AD = axillary dissection

S = lymph node sampling

Goyal *et al*., in the ALMANAC trial retrospectively evaluated the accuracy of the technique in 75 patients, finding a 8.8% false negative rate. However, SLNB is compared with node sampling instead of a complete AD in most patients and diagnosis of MF-MC cancer was retrospectively obtained only at final pathologic specimens [37].

More recently, a large prospective multi-institutional trial of the Austrian Sentinel Node Study Group was reported by Knauer *et al*., [38]. In this study the false negative rate of SLNB in 150 MC cancer, 125 of whom underwent AD, was 4.1%, not too far from the one reported in our study.

The prospective monoinstitutional series published in the literature, although performed in small patients populations [30,31,11,33], report excellent results in all but one study [33], where Ozmen *et al*., show a 33% false negative rate in MF breast cancers. However, the value of this study is limited by the fact that only MF cancers were enrolled (MC tumors were excluded) and the majority of the cases of this series (18 of 21) had only retrospective, pathologic diagnosis of multifocality.

A possible explanation for this unique unacceptable result, it may be due to technical reasons related to the use of blue dye alone as tracer, injected through peritumoral route, with a very low success rate in SLN identification (85.7%). The Authors can only conclude that "SLNB using peritumoral blue-dye injection method is not reliable to be performed in patients with MF disease".

In our experience both areolar (A) and double intradermal (2ID: over the two dominant tumors) injection techniques have demonstrated equally feasible in MF-MC tumors: no differences have been shown in success rate, lymphoscintigraphic anatomy and mean number of SLNs identified. However, 2 ID technique was used mainly for the lymphoscintigraphic study aimed to evaluate the route of lymphatic spreading from different sites of the breast. Once demonstrated that both pathway converged into one sentinel lymphatic channel in over 90% of cases, it appears useless and time-consuming to use more than one site of injection (with double dose of radioactivity).

Although a single ID injection over the largest-size lesion has been used in others' experience in case of MF-MC tumors with similar results, areolar injection has the further advantage of being independent from the location of the nodules, which facilitates the procedure, especially in case of non-palpable lesions.

To date our series is the largest monoinstitutional prospective study on SLNB in MF-MC invasive breast cancer and the high accuracy (96.8%) is comparable to other differently designed series. Moreover, since surgeons are aware of the possibility of a skip metastasis due to neoplastic obstruction of lymphatic vessels afferent to the true SLN [16,17], during a routine procedure of SLNB a non radioactive but suspicious LN would be removed and frozen sections could reveal the positive status of axilla. On this basis, if the contraindication to SLNB due to clinically N+ finding is considered both for preoperative and intraoperative diagnosis, the case of skip metastases identified by the surgeon in our study has to be considered excluded from the study, giving a FN rate of 0%, and all sensitivity, negative predictive value and accuracy of 100%.

## Conclusion

Our lymphoscintigraphic study demonstrated that axillary SLN represents the whole breast regardless of tumor location within the parenchyma. MF and MC breast cancers should not be considered anymore a contraindication for the SLNB technique. Radioisotope injection through areolar route can be proposed as the best option mapping technique for MF-MC tumors.
